# Cardiomyocyte Transplantation after Myocardial Infarction Alters the Immune Response in the Heart

**DOI:** 10.3390/cells9081825

**Published:** 2020-08-03

**Authors:** Praveen Vasudevan, Markus Wolfien, Heiko Lemcke, Cajetan Immanuel Lang, Anna Skorska, Ralf Gaebel, Dirk Koczan, Tobias Lindner, Robby Engelmann, Brigitte Vollmar, Bernd Joachim Krause, Olaf Wolkenhauer, Hermann Lang, Gustav Steinhoff, Robert David

**Affiliations:** 1 Department of Cardiac Surgery, Rostock University Medical Centre, 18057 Rostock, Germany; Praveen.Vasudevan@med.uni-rostock.de (P.V.); Heiko.Lemcke@med.uni-rostock.de (H.L.); anna.skorska@med.uni-rostock.de (A.S.); Ralf.Gaebel@med.uni-rostock.de (R.G.); Gustav.Steinhoff@med.uni-rostock.de (G.S.); 2 Department of Life, Light and Matter, University of Rostock, 18059 Rostock, Germany; 3 Department of Operative Dentistry and Periodontology, Rostock University Medical Centre, 18057 Rostock, Germany; hermann.lang@med.uni-rostock.de; 4 Department of Systems Biology and Bioinformatics, Institute of Computer Science, University of Rostock, 18057 Rostock, Germany; markus.wolfien@uni-rostock.de (M.W.); olaf.wolkenhauer@uni-rostock.de (O.W.); 5 Department of Cardiology, Rostock University Medical Centre, 18057 Rostock, Germany; Cajetan.Lang@med.uni-rostock.de; 6 Core Facility for Microarray Analysis, Institute for Immunology, Rostock University Medical Centre, 18057 Rostock, Germany; dirk.koczan@med.uni-rostock.de; 7 Core Facility Multimodal Small Animal Imaging, Rostock University Medical Centre, 18057 Rostock, Germany; Tobias.Lindner@med.uni-rostock.de; 8 Core Facility for Cell Sorting & Cell Analysis, Laboratory for Clinical Immunology, Rostock University Medical Centre, 18057 Rostock, Germany; robby.engelmann@med.uni-rostock.de; 9 Rudolf-Zenker-Institute for Experimental Surgery, Rostock University Medical Centre, 18057 Rostock, Germany; Brigitte.Vollmar@med.uni-rostock.de; 10 Department of Nuclear Medicine, Rostock University Medical Centre, 18057 Rostock, Germany; Bernd.Krause@med.uni-rostock.de; 11 Stellenbosch Institute of Advanced Study (STIAS), Wallenberg Research Centre at Stellenbosch University, Stellenbosch 7602, South Africa

**Keywords:** infarction, cardiomyocytes, transplantation, immune response, translation

## Abstract

We investigated the influence of syngeneic cardiomyocyte transplantation after myocardial infarction (MI) on the immune response and cardiac function. Methods and Results: We show for the first time that the immune response is altered as a result of syngeneic neonatal cardiomyocyte transplantation after MI leading to improved cardiac pump function as observed by magnetic resonance imaging in C57BL/6J mice. Interestingly, there was no improvement in the capillary density as well as infarct area as observed by CD31 and Sirius Red staining, respectively. Flow cytometric analysis revealed a significantly different response of monocyte-derived macrophages and regulatory T cells after cell transplantation. Interestingly, the inhibition of monocyte infiltration accompanied by cardiomyocyte transplantation diminished the positive effect of cell transplantation alone. The number of CD68+ macrophages in the remote area of the heart observed after four weeks was also different between the groups. Transcriptome analysis showed several changes in the gene expression involving circadian regulation, mitochondrial metabolism and immune responses after cardiomyocyte transplantation. Conclusion: Our work shows that cardiomyocyte transplantation alters the immune response after myocardial infarction with the recruited monocytes playing a role in the beneficial effect of cell transplantation. It also paves the way for further optimization of the efficacy of cardiomyocyte transplantation and their successful translation in the clinic.

## 1. Introduction

Cardiovascular diseases are a major cause of morbidity and mortality in the world [1]. Myocardial infarction (MI) is caused due to insufficient blood supply and ischemia leading to progressive heart muscle damage and cardiac remodeling with a high risk of mortality. There is a variety of treatment options available ranging from pharmacological treatments, small molecules that stimulate endogenous regeneration, cell transplantation and immune modulation to heart transplantation. Their potential to regenerate the ailing myocardium and vast troves of previously published literature has made stem cell transplantation a dominant candidate as a preclinical and clinical treatment strategy for MI [2]. The administration of bone marrow-derived stem cells and stem cells from other sources to thousands of patients over many years has failed to yield their primary efficacy end point. The marginal benefit of these cell transplantation studies has been attributed to diverse reasons with no clear scope of improvement owing mainly to the lack of clarity on their mechanism of action. This roadblock has led to people questioning the future of stem cell therapy as a viable treatment option [3].

The future of regenerative medicine and cardiac cell therapy also foresee the emergence of pluripotent stem cell-derived cardiomyocytes as a better choice of cell type for transplantation, because these cells have been shown to graft successfully and regenerate infarcted hearts [4]. There is a clear lack of studies available on the interaction of transplanted cardiomyocytes with the inflammatory pathway under ischemic conditions. The exact mechanism of action of these transplanted cells is also not entirely defined. This fundamental gap should be first uncovered in order to provide a comprehensible path for the successful implementation of newer pluripotent stem cell-derived cell therapies in the clinic.

In recent years, we have seen the emergence of a new wave of therapeutic strategies for treating MI by interventional immune therapy. The various immune cells are highly important for cardiac regeneration and the subsequent healing process after MI [5]. Modulating the various cell macrophage subpopulations after MI has been shown to contribute to improved cardiac function along with a smaller scar size [6]. Incidentally, the beneficial effects of syngeneic mesenchymal stem cell (MSC) transplantation have been shown to be attributed to the transition to an anti-inflammatory macrophage subset [7]. They have also been shown to modulate T cells [8], B cells [9] and Natural killer (NK) cells [10]. Thus, it is evident that there is a clear cross talk between the host immune response and the transplanted cells.

In accordance with the described background, this study aims to investigate the impact of intramyocardial syngeneic neonatal cardiomyocyte transplantation after MI on cardiac function and the various immune subpopulations in the heart. Induced pluripotent stem cell (iPSC)-derived cardiomyocytes represent an ideal cell source for cardiac regeneration. However, there is no consensus on which differentiation protocol and cell stage are most suitable for eliciting a beneficial effect after transplantation [11,12], while the immunogenicity of the cells at the various differentiation stages is also not known. Therefore, the transplantation of syngeneic neonatal cardiomyocytes allows us to reproducibly study the beneficial effects of cardiomyocytes without the involvement of a transplant rejection response by the host immune system obscuring the effect of cell transplantation on the immune response. Our study seeks to elucidate the mechanism of action of transplanted cardiomyocytes by investigating the effect of these cells after MI in wildtype C57BL/6J mice.

## 2. Materials and Methods

### 2.1. Mice

8–12 weeks old C57BL/6J mice (Charles River, Wilmington, MA, USA) were used for this study and were bred in the animal facility of the Rostock University Medical Center and maintained in specified pathogen-free conditions with food and water ad libitum prior to use. All animal experiments were approved by the local animal care committee of the “Landesamt für Landwirtschaft, Lebensmittelsicherheit und Fischerei Mecklenburg-Vorpommern” (LALLF, Rostock, Germany) (approval number. LALLF M-V/TSD/7221.3-1-015/15) and conducted according to EU guidelines on animal protection.

### 2.2. Neonatal Cardiomyocyte Isolation

Ventricles were harvested from neonatal transgenic B6.e GFP mice after decapitation, minced, and single-cell suspensions were prepared using the Pierce primary cardiomyocyte isolation kit (Thermo Fisher Scientific, Waltham, MA, USA). 1 × 10^6^ cells were then suspended in 15 µL Matrigel^TM^ (Corning, Berlin, Germany) for injection.

### 2.3. Surgical Procedure

**Figure 1:**
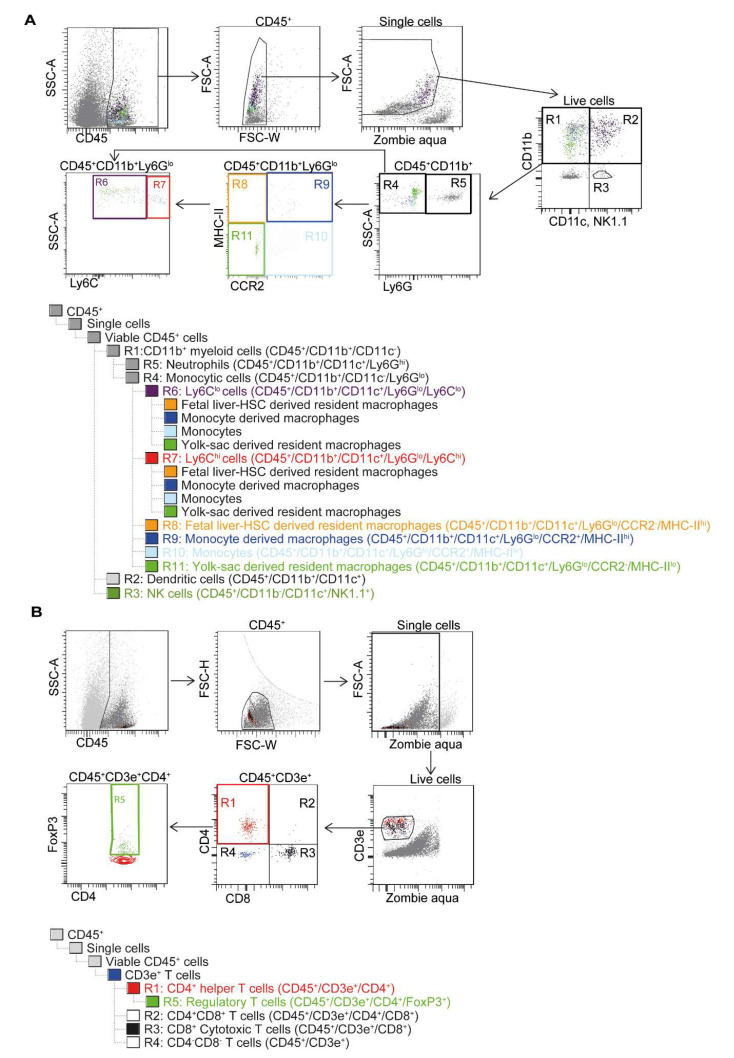
Gating strategy for identifying the specific immune subpopulations using Flow cytometry. Firstly, the mononuclear cells (MNC) that express CD45 were gated and only single cells were included into the analysis. Dead cells were excluded using Zombie-AmCyan dye. The remaining viable CD45^+^ MNCs were further characterized based on the expression of specific markers using two different panels: (**A**) Myeloid panel, (**B**) T cell panel. In both cases, only CD45 expressing mononuclear single-cell suspensions was included. In the myeloid panel, the live cells were then grouped based on their specific marker expression: R1: CD11b+ myeloid cells, R2: Dendritic cells, R3: NK cells, R4: Monocytic cells, R5: Neutrophils, R6: Ly6C^hi^ or commonly known as M1 cells, R7: Ly6C^lo^ or commonly known as M2 cells, R8: Fetal liver-HSC derived resident macrophages, R9: Monocyte derived macrophages, R10: Monocytes, R11: Yolk-sac derived resident macrophages. In the T cell panel, the viable CD45^+^ cells were then gated for T cells based on the expression of CD3e. The T cells were then further grouped based on their specific marker expression combinations: R1: CD4^+^ helper T cells, R2: CD4^+^ CD8^+^ T cells, R3: CD8^+^ Cytotoxic T cells, R4: CD4^−^ CD8^−^ T cells.

Mice were anesthetized with intraperitoneal administration of Pentobarbital (50 mg/kg body weight) and subcutaneous administration of Fentanyl (2 µg/kg body weight). The experimental setup is shown in Figure 1A. Briefly, they were intubated and the thorax was opened. Following thoracotomy, permanent myocardial infarction was induced through ligation of the left anterior descending coronary artery (LAD). The suture was left in the heart without ligation in the Sham group. Three days after the initial thoracotomy, either 1 × 10^6^ neonatal GFP cardiomyocytes suspended in 15 µL Matrigel^TM^ (MIC and MIC+I) or only Matrigel^TM^ (Sham and MI) were injected intramyocardially in four separate injections around the peri-infarct border zone. Following these procedures, the thorax was closed and the skin was stitched using a resorbable suture. Vetalgin (MSD animal health GmbH, Lucerne, Switzerland) was administered as an analgesic by mixing it in the drinking water. In the MIC+I group, the inhibition of monocyte infiltration into the heart was carried out with the oral administration of a CCR2 inhibitor (RS504393; Sigma-Aldrich, Saint Louis, MO, USA) twice daily (2 mg/kg bodyweight) for five days (day 3 to day 7 post MI) accompanied by cell transplantation. Following the completion of the experiment, the mice were euthanized by cervical dislocation and the organs were harvested for further analysis.

### 2.4. Isolation of Mononuclear Cells for Flow Cytometry

Cardiac mononuclear cell suspensions were prepared as previously described [13]. Briefly, the ventricles were dissected and enzymatically digested in a collagenase enzyme mixture for 20 min at 37 °C. Splenic mononuclear cell suspensions were prepared simply by mechanically shearing the tissue using a syringe. The digest was then filtered to remove undigested tissue, centrifuged, treated with RBC lysis buffer and resuspended in MACS^®^ buffer (Phosphate buffer saline, 2 mM Ethylenediaminetetraacetic acid, 0.5% Bovine serum albumin) for further staining and analysis using flow cytometry.

### 2.5. Flow Cytometry with Gating Strategy

Mononuclear cells isolated from the tissues were labelled with Zombie Aqua dye (BioLegend, San Diego, CA, USA) for live–dead staining, washed, resuspended in MACS buffer with FCR block (Miltenyi Biotech GmbH, Bergisch Gladbach, Germany) and stained further with the antibodies listed in Table 1. Cardiac mononuclear cells were stained using the antibodies described in the myeloid panel (Figure 1A) and the T cell panel (Figure 1B), while the splenic cells were stained for only the T cell panel (Figure 1B). The stained samples were analyzed on BD FACS LSR II^®^ running BD FACS Diva software (Becton Dickinson, Franklin Lakes, NJ, USA), and the various immune cell subpopulations were assessed using the gating strategy described in (Figure 1).

### 2.6. Magnetic Resonance Imaging (MRI)

Magnetic resonance imaging (MRI) measurements of the left ventricular ejection fraction (LVEF) were performed after four weeks on a small animal MRI system (BioSpec 70/30, 7.0 T magnetic field strength, maximum gradient strength 440 mT/m, Bruker BioSpin GmbH, Ettlingen, Germany) equipped with a ^1^H transmit volume coil (86 mm, volume resonator) and a 2-by-2 receive-only surface coil array (both Bruker BioSpin GmbH). Anesthesia was induced using 2%–3.5% isoflurane in oxygen gas provided by an animal anesthesia device (Univet porta, Groppler Medizintechnik, Deggendorf, Germany). Animals were placed on a dedicated mouse bed in the supine position and a surface coil was placed on the thorax of the mice. Body temperature and respiration rate were monitored using an MR-compatible small animal monitoring and gating system (Model 1030, SA Instruments, Inc., Stony Brook, NY, USA) and stable body temperature was maintained by warm water heating. Anesthesia was maintained during the experiment with isoflurane–oxygen (1.5%–2%) to achieve a respiration rate of about 35 to 55 breaths. After several planning sequences for the short axes view, final images for the left ventricular ejection fraction (LVEF) measurements were acquired using IntraGate gradient-echo cine sequences (IntraGate Cine-FLASH) in six short-axis planes completely covering the left ventricle (LV). The acquisition parameters were as follows: echo time (TE): 2.38 ms, repetition time (TR): 5.89 ms, flip angle: 15°, 14 frames per cardiac cycle, oversampling: 140, averages: 1, field of view (FOV): 29.4 × 25.2 mm, matrix size: 211 × 180, resolution in-plane: 0.14 × 0.14 mm, slice thickness: 1 mm, scan time per slice: 2 min.

Data analysis was subsequently carried out using the freely available Segment v2.0 software R5165 [14]. The LV endocardium along five planes of the LV was manually segmented and integrated to yield the volumetric parameters.

### 2.7. Conductance Catheter

Four weeks after surgery, the mice were anesthetized and the pressure characteristics of the left ventricle were assessed using the Millar Pressure-Volume System (Ultra-Miniature Pressure-Volume Catheter (model SPR-1030), Millar Pressure Conductance Unit (model MPCU-200) and Millar PowerLab data-acquisition hardware from Emka Technologies (Paris, France). The pressure was calibrated by equating the minimal and maximal conductance with the minimal (0 mmHg) and maximal (100 mmHg) pressures from venous circulation. Retrograde access to the left ventricle was attained through cannulation of the carotid artery. The pressure data were analyzed using IOX Version 1.8.3.20 software (Emka Technologies). Following the measurements, the hearts were arrested in the diastolic phase by administration of 5% KCl. The hearts were then removed and flash frozen in Tissue-Tek^®^ O.C.T. Compound (Sakura Finetek, Alphen aan den Rijn, The Netherlands) with liquid nitrogen.

### 2.8. Histological Analysis

Frozen hearts were cryosectioned into 6 µm axial sections divided into five different levels from the apex to the base. For fibrosis estimation, the heart sections were stained with Fast Green FCF (Sigma-Aldrich) and Sirius Red (Chroma Waldeck GmbH & Co. KG, Münster, Germany). The relative fibrotic area (Sirius Red positive area/whole left ventricle area) of two contiguous levels was then quantitatively assessed using computerized planimetry (Axio Vision LE Rel. 4.5 software, Carl Zeiss AG, Oberkochen, Germany).

### 2.9. Immunofluorescence Staining

For immunostaining, the sections were fixed in buffered 4% Paraformaldehyde (Sigma-Aldrich) for 15 min at room temperature. They were then blocked for one hour in DAKO protein block (Agilent Technologies, Santa Clara, CA, USA) to reduce unspecific binding. For labelling endothelial cells, the sections were stained overnight at 4 °C d with goat anti-CD31 (Abcam, Cambridge, UK) for detecting endothelial cells, rat anti-CD68 (Bio-Rad Laboratories, Hercules, CA, USA) for macrophage detection and with anti-GFP (Abcam) for detecting GFP cells. They were subsequently incubated with AlexaFluor conjugated secondary antibodies (Thermo Fisher Scientific) for one hour at 37 °C. After washing, the sections were mounted on cover slips with DAKO mounting medium containing DAPI (Agilent Technologies). Fluorescence images were obtained using a Zeiss ELYRA PS.1 LSM 780 confocal imaging system (Carl Zeiss AG).

### 2.10. Microarray

RNA integrity was evaluated using the Agilent Bioanalyzer 2100 with the RNA 6000 Nano kit (Agilent Technologies). For microarray analysis, 200 ng of isolated RNA samples was applied and hybridization was performed as described previously [15]. Hybridization was carried out on Affymetrix Clariom^TM^ D Arrays according to the instructions of the manufacturer (Thermo Fisher Scientific).

### 2.11. Microarray Analysis

The microarray data analysis was conducted with the provided Transcriptome Analysis Console Software from Thermo Fischer Scientific (Version 4.0.1). The analysis included quality control, data normalization and statistical testing for differential expression (Limma). Transcripts are considered as significantly differentially expressed with a two-fold change (FC) higher or lower, false discovery rate (FDR) < 0.5 and *p*-value < 0.05. Based on the annotated biological processes and molecular functions of the differentially expressed transcripts described by the Gene Ontology (GO) terms, we created our gene expression networks to show the connection and significance of each GO term using the Cytoscape application ClueGo [16]. Heatmaps were generated using heatmapper [17].

### 2.12. Statistics

All data are presented as the mean values ± standard error of the mean (SEM). A Student’s *t*-test was used for statistical analysis of normally distributed data and a Mann–Whitney test was used for a non-parametric analysis of flow cytometric data. Microarray data were tested using Limma. Values of *p* < 0.05 were considered as statistically significant.

## 3. Results

### 3.1. Cardiomyocyte Transplantation Alters the Dynamics of the Immune Response in the Heart after MI in C57BL/6J Mice

**Figure 2:**
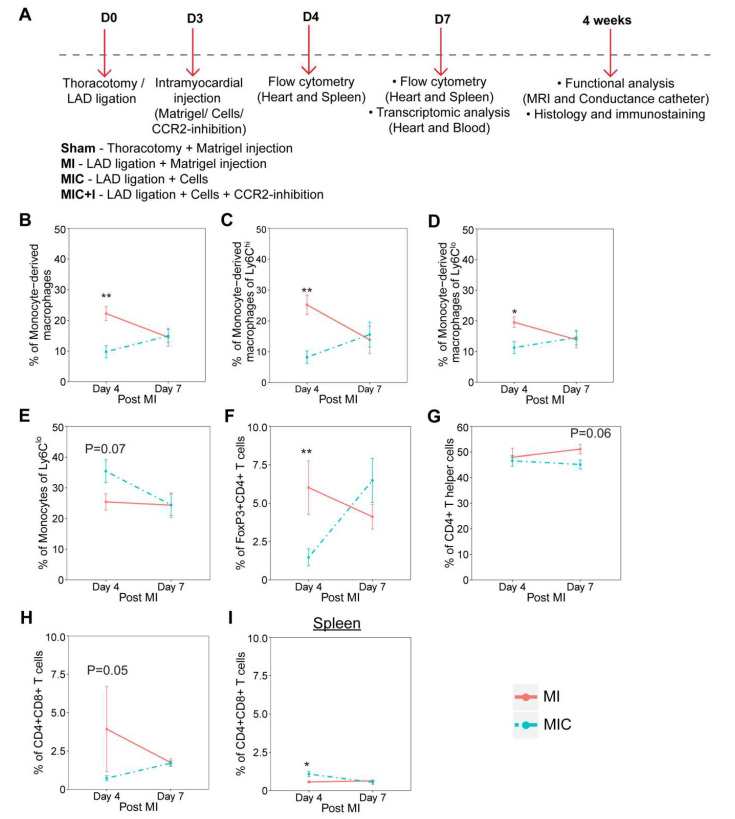
Cardiomyocyte transplantation alters the immune response in the heart after myocardial infarction (MI). (**A**) Experimental setup. (**B**–**I**) Flow cytometric analysis of the various immune cell populations in the heart (**B**–**H**) and spleen (**I**) of C57BL/6J mice four and seven days following MI and cardiomyocyte transplantation (MIC). The various cell populations were identified based on the strategy presented in Figure 1. *n* = 7. Values are represented as the mean ± SEM. Significance was calculated using the Mann–Whitney test. * *p* < 0.05, ** *p* < 0.01.

Mice underwent permanent MI through ligation of the LAD. Three days after MI, either 1 × 10^6^ neonatal GFP cardiomyocytes suspended in 15 µL Matrigel^TM^ (MIC) or only Matrigel^TM^ (MI) were injected intramyocardially. We observed a significant decrease in the percentage of monocyte-derived macrophages (Figure 2B) in the heart, with a corresponding decrease also in the contribution of monocyte-derived macrophages to the Ly6C^hi^ (Figure 2C) and Ly6C^lo^ populations (Figure 2D), with an increase in the percentage of monocytes contributing to the Ly6C^lo^ pool in the heart (Figure 2E) four days after MI in the cardiomyocyte treated group compared to the MI control. Interestingly, we did not find any differences in the percentage of proinflammatory or anti-inflammatory macrophages and monocytes between the cell treated and MI control groups.

In the lymphoid based contribution to the immune response, we observed a significant reduction in the percentage of CD4^+^FoxP3^+^ T cells (Figure 2F), commonly referred to as T_reg_ cells and CD4^+^CD8^+^ T cells (Figure 2H) in the heart with a coincidental increase in the percentage of CD4^+^CD8^+^ T cells in the spleen (Figure 2I) four days after MI in the cardiomyocyte treated group compared to the MI control. There was a slight reduction in the percentage of CD4^+^ T helper cells (Figure 2G) in the heart seven days after MI in the cardiomyocyte treated group compared to the MI control. It should be mentioned that we were able to assess only a low frequency of CD4^+^ T cells and even fewer events of T_reg_ cells owing to their rarity of occurrence in the heart notwithstanding their important role in regulating the immune response with these numbers.

### 3.2. Intramyocardial Syngeneic Cardiomyocyte Transplantation Improves Cardiac Pump Function

**Figure 3:**
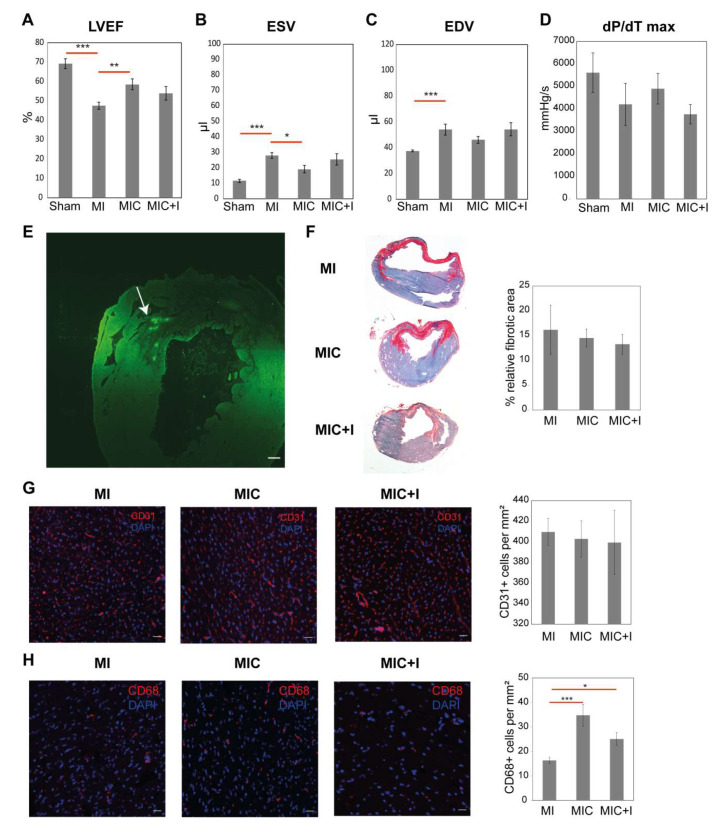
Syngeneic cardiomyocyte transplantation after MI leads to improved cardiac pump function and macrophage infiltration with no improvement in fibrosis and capillary density. Assessment of (**A**) Left Ventricular Ejection Fraction (LVEF,%), (**B**) End Systolic Volume (ESV, µL), (**C**) End Diastolic Volume (EDV, µL) using MRI (*n* = 7) and pressure characteristics, (**D**) dP/dT max (mmHg/s) using conductance catheter (*n* = 6–7) four weeks after MI. (**E**) A tile scan of the heart four weeks after cell transplantation with an arrow pointing towards GFP signals observed at the injection site. Scale bar represents 200 µm. (**F**) Assessment of fibrotic area in the heart after four weeks using Fast Green/Sirius Red staining (*n* = 7). The heart slices were observed using a 10× objective. (**G**) Assessment of CD31^+^ cells in the remote area of the heart four weeks after MI (*n* = 7). CD31^+^ cells were stained red and the nuclei were stained blue using DAPI. Scale bar represents 20 µm. (**H**) Assessment of CD68^+^ cells in the remote area of the heart four weeks after MI (*n* = 7). CD68^+^ cells were stained red and the nuclei were stained blue using DAPI. Scale bar represents 20 µm. Values are represented as the mean ± SEM. Significance was calculated using the Student‘s *t*-test and Mann–Whitney test. * *p* < 0.05, ** *p* < 0.01, *** *p* < 0.001.

Cardiac morphology and function were assessed four weeks after MI/thoracotomy using MRI. The pressure characteristics were also recorded after MRI using a conductance catheter. Cardiomyocyte transplantation led to a significant improvement in cardiac function as observed by the increase in LVEF (58.57% ± 2.83% vs. 47.57% ± 1.77%, *p* = 0.006) (Figure 3A), decreased ESV (19.17 ± 2.41 µL vs. 28 ± 1.90 µL, *p* = 0.017) (Figure 3B) and lowered but not significant End Diastolic Volume (EDV) (46.17 ± 2.65 µL vs. 54.14 ± 4.22 µL) (Figure 3C) when compared to the MI group. We observed only a marginal improvement in the dP/dT max values (4900.97 ± 552.55 mmHg/s vs. 4220.44 ± 527.72 mmHg/s) (Figure 3D). We were also able to observe GFP signals signifying the transplanted cells at the injection site four weeks after cardiomyocyte transplantation in the heart (Figure 3E).

The fibrotic area was then calculated from two different levels using Sirius Red staining (Figure 3F). Cell therapy did not lead to a significant decrease in the size of the fibrotic scar (14.53% ± 1.82% vs. 16.19% ± 4.95%) when compared to the infarct control in C57BL/6J mice. It should be noted that the relatively small infarcts were in agreement with the LVEF values.

Contrary to most of the cell transplantation studies showing an increase in capillary density, we observed no such improvement in capillary density in the remote area four weeks after cardiomyocyte transplantation as assessed by CD31 staining (Figure 3G).

### 3.3. CCR2 Inhibition Diminishes the Positive Effect of Cardiomyocyte Transplantation Alone

The inhibition of monocyte infiltration into the heart using a CCR2 inhibitor has been previously shown to be beneficial [6]. In order to further augment the efficiency of cardiomyocyte transplantation and prolong the inhibition of CCR2^+^ macrophages as observed one day after transplantation (Figure 2A), we sought to combine the transplantation of cardiomyocytes accompanied by oral administration of the CCR2 inhibitor (RS504393) for five days (MIC+I).

Contrary to our expectations, the improvement in LVEF (54% ± 3.42% vs. 47.57% ± 1.77%) (Figure 3A) and ESV (25.43 ± 3.64 µL vs. 28 ± 1.90 µL) (Figure 3B) four weeks after infarction, when compared to the MI group, was smaller in comparison to the cell transplanted group. The EDV (54.29 ± 5.12 µL vs. 54.14 ± 4.22 µL) (Figure 3C) and dP/dT max values (3762.73 ± 325.64 mmHg/s vs. 4220.44 ± 527.72 mmHg/s) (Figure 3D) were even marginally worse that the MI control group. The improvement in cardiac function was not significant anymore. There was also no significant improvement in the relative fibrotic area (Figure 3F). Similarly, there was also no significant difference in the endothelial numbers observed in the remote area of the heart (Figure 3G) using CD31^+^ staining.

### 3.4. CD68^+^ Macrophage Numbers in the Remote Area of the Heart Varies between the Groups

We proceeded to quantify the number of CD68^+^ macrophages in the remote area of the heart (Figure 3H) after four weeks to ascertain if their numbers are also affected by cardiomyocyte transplantation. Interestingly, we found that there was almost 2.5 times more CD68^+^ cells in the remote area of the cell treated group (34.77 ± 4.47 vs. 16.36 ± 1.25, *p* = 0.002) when compared to the infarct group. With CCR2-inhibition, there was still a significantly higher number of CD68^+^ cells in the remote area of the heart in the MIC+I group (25.08 ± 2.66 vs. 16.36 ± 1.25, *p* = 0.012) when compared with the MI group albeit fewer than in the MIC group.

### 3.5. Transcriptomic Profiling of the Heart and Blood Revealed Striking Differences in Gene Expression Due to Cardiomyocyte Transplantation

**Figure 4:**
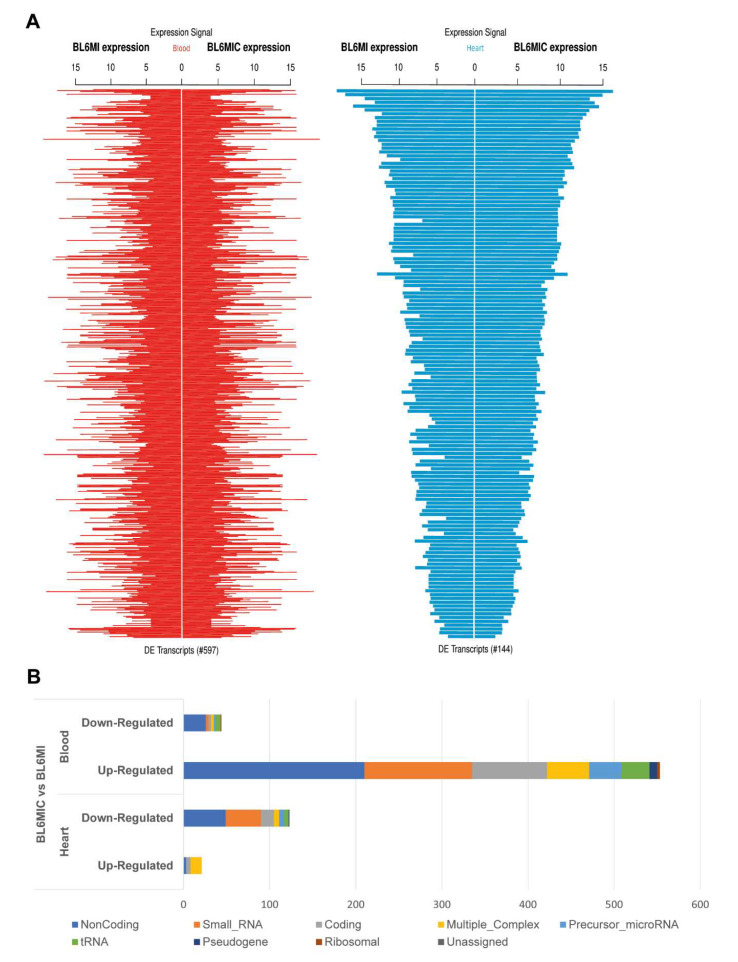
Transcriptomic analysis of the heart and blood. C57BL/6J mice were subjected to MI, MIC and seven days later, the RNA from the heart and blood was isolated and analyzed via a Clariom^TM^ D microarray. (**A**) The log(2) scale expression signals of the differentially expressed (DE) transcripts between the cell treated (BL6MIC) and infarct (BL6MI) groups are illustrated as Manhattan plots. DE transcripts in the blood are shown in red while the DE transcripts from the heart are shown in blue. Transcripts are considered DE, if they show a two-fold change and have a *p*-value < 0.05. (**B**) The relative contribution of the various categories of RNA to these differentially expressed transcripts in the heart and blood (*n* = 3).

We assessed the transcriptional profile of the heart and blood of MI and MIC groups seven days after MI via Clariom^TM^ D microarray. The subsequent analysis of these samples revealed several hundred differentially expressed transcripts (two-fold or greater with *p* < 0.05) with more overall differentially expressed transcripts in the blood (Figure 4A). The relative contribution of the various RNA categories (Figure 4B) to these differential expressed targets was also heterogeneous with a surprisingly substantial contribution from non-coding RNA (ncRNA). Transcription factors are marked with a ‘*’ next to their name.

### 3.6. Cardiomyocyte Transplantation in C57BL/6J Mice Primarily Influences the Circadian Regulation and Mitochondrial Metabolism

**Figure 5:**
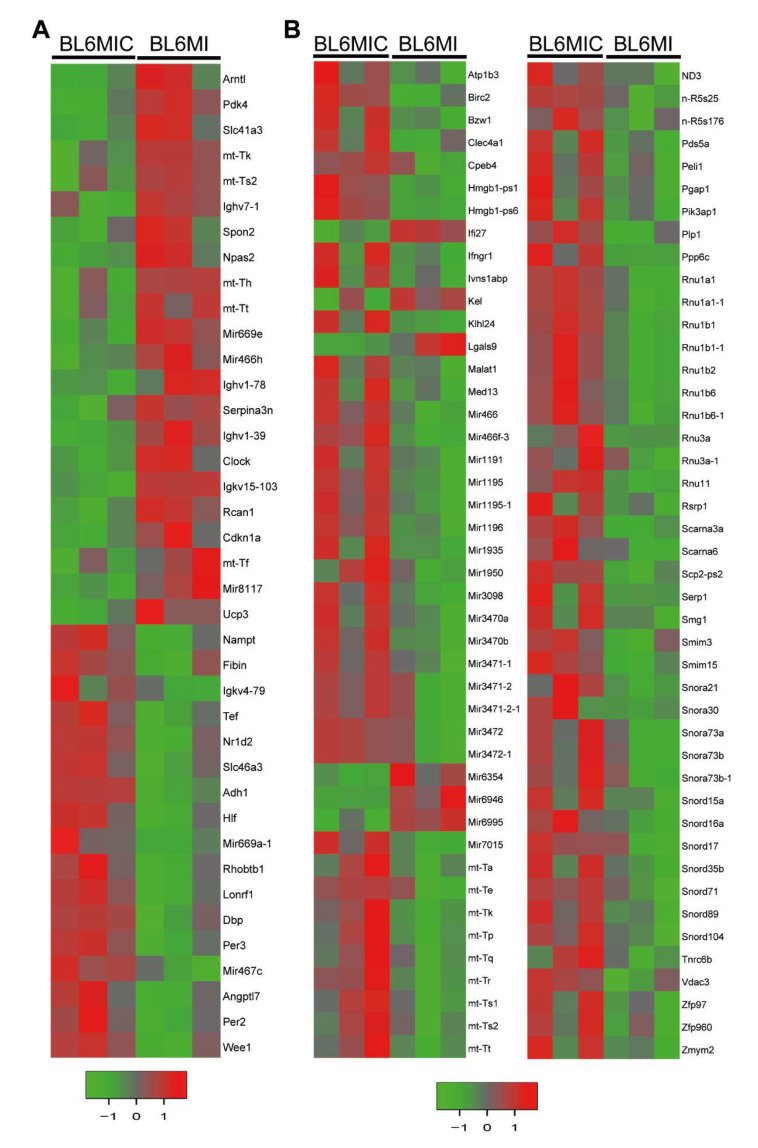
Cardiomyocyte transplantation has varied effects on the gene expression in the heart and blood. Heatmap of annotated differentially expressed (DE) transcripts found between BL6MIC and BL6MI in the heart (**A**) and blood (**B**). Transcripts are considered DE if they show a two-fold change and have a *p*-value < 0.05. Upregulated targets are colored red while the downregulated targets are colored green.

Transplantation of cardiomyocytes in C57BL/6J mice appeared to manifest its effects primarily through its effects on the transcripts expressed in circadian regulation and mitochondrial metabolism in the heart (Figure 5A) and blood (Figure 5B). The circadian genes that are either up or down regulated in the heart include Arntl*, Cdkn1a, Clock*, Dbp*, Npas2*, Nr1d2*, Per2, Per3, Tef* and Ucp3. Interestingly, expression of pyruvate dehydrogenase kinase (Pdk4), Slc41a3 and several mitochondrial tRNAs was significantly reduced in the MIC heart group. There was also an increase in ANGPTL-7 expression, which has been shown to promote angiogenesis [18]. The immune response in the heart was also affected by the altered expression of various immunoglobulins and lowered Spon2 in the cell transplanted group. Expression of Rcan1, which is involved in cardiac hypertrophy, was reduced in the MIC group. Contrastingly, the mitochondrial tRNAs and ND3, which are responsible for oxidative phosphorylation in mitochondria, were upregulated in the blood in MIC mice. Additionally, several small RNAs involved in mRNA processing in the blood were upregulated. Interestingly, the expression of Lgals9, which is involved in immune cell interaction and activity, was downregulated in the blood of MIC animals. IFNGR1 and Birc2 targets, that are involved in focal adhesion, were also upregulated in the blood. The entire gene expression machinery activation in the blood could be attributed to the increased expression of Med13 in the blood.

**Figure 6:**
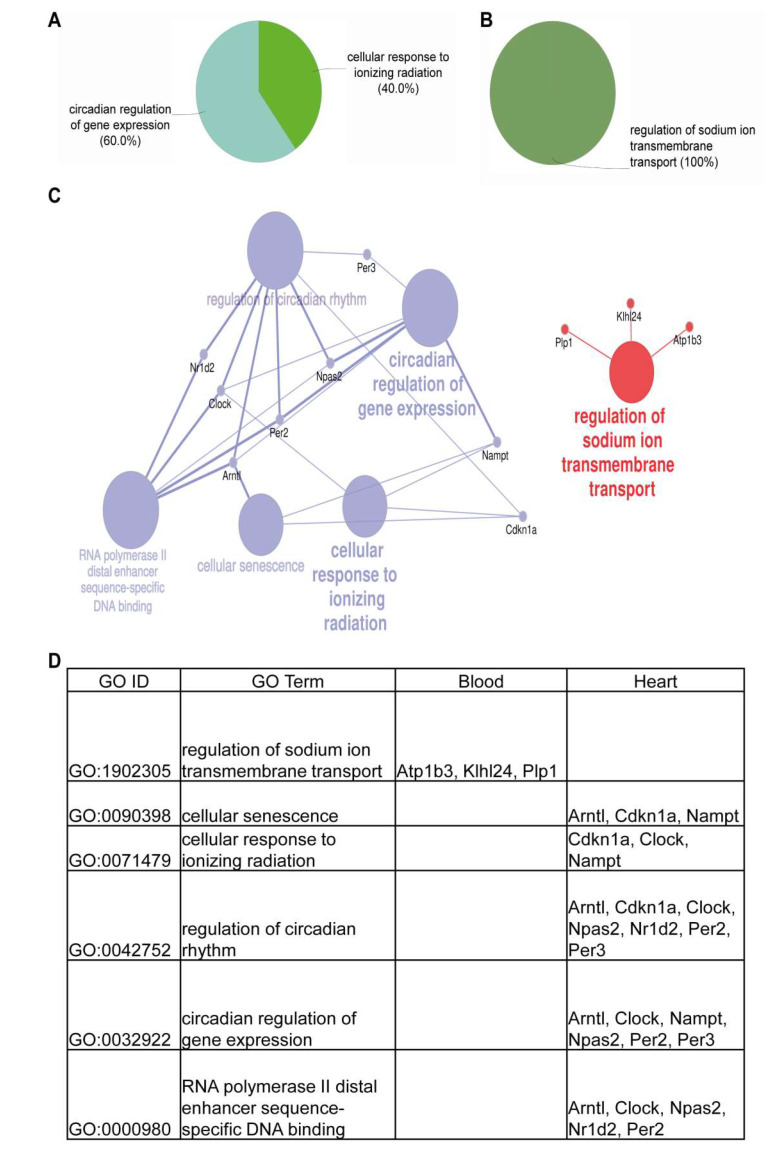
Gene Ontology (GO) enrichment and network analysis reveals the pathways influenced by cardiomyocyte transplantation. GO enrichment analysis expressed as % terms per group was performed on differentially expressed (DE) targets in the heart (**A**) and blood (**B**). (**C**) Network analysis using the GO terms of these targets. In the network, the transcripts from the heart and the corresponding GO terms are represented in blue circles with the transcripts from the blood and their corresponding GO terms being represented in red circles. (**D**) The respective GO ID and GO terms of these DE targets in the heart and blood.

The GO enrichment analysis of the differentially expressed (DE) transcripts in the heart and blood of C57BL/6J mice (Figure 6A–D) showed only a limited number of pathways, which were distinct in the heart and blood. Network enrichment analyses and visualization (Figure 6C) of these DE transcripts showed how closely these transcripts are involved in circadian regulation and also in transcription through RNA polymerase II binding in the heart and sodium ion transmembrane transport regulation in the blood. These networks seemed to be distinctly available in the heart and blood with no available crosslink.

## 4. Discussion

Our current study shows for the first time that cardiomyocyte transplantation alters the immune response in the heart and that the inflammatory response is important in deciding the functional outcome of cardiomyocyte transplantation.

Cardiomyocyte transplantation reduces the percentage of CCR2^+^ monocyte-derived macrophages along with T_reg_ cells during the acute MI response in the heart as observed using flow cytometry (Figure 2) while also having long term effects on the prevalence of macrophages as evidenced by the increased CD68 numbers in the remote area of the heart four weeks after MI (Figure 3H). The effect of cardiomyocyte transplantation on the immune response is therefore long lasting encompassing both the acute and chronic MI phases. The contributions of the various monocyte and macrophage subpopulations to the increased CD68 numbers, however, cannot be established without suitable lineage tracing studies.

Previous studies with early depletion of the CCR2^+^ cardiac macrophages have been shown to result in decreased left ventricular systolic function [19] while CCR2^+^ monocytes have been shown to be almost entirely pro-inflammatory [20]. This is in accordance with the hypothesis put forward by Dick et al. [19] who suggest that inhibiting monocyte influx could impair later development of monocyte-derived macrophages with reparative functions. Interestingly, we observed that cardiomyocyte transplantation accompanied by prolonged CCR2 inhibition diminishes the cardiac functional improvement observed with the cells alone. Recent single cell sequencing data revealed a continuum of states of blood borne monocyte-derived macrophages ranging from pro-inflammatory to anti-inflammatory across the injury response [21]. This shows how the complexity and plasticity of these macrophages and the duration and timing of their modulation lead to different outcomes. Therefore, a more thorough understanding of the influence of transplanted cells on the immune response would pave the way for optimizing their time of administration. While most of the cell transplantation studies were carried out immediately after MI [22,23,24,25,26] or one week after MI [27,28,29,30], our idea of transplanting the cells three days after MI offers an opportunity to influence the immune response during its transition from the pro-inflammatory to the anti-inflammatory state. Understanding the exact nature and status of these immune cells and cues to influence this transition is therefore important in strategizing the timing of cell transplantation.

Studies utilizing highly sophisticated differentiation protocols and engineered tissue matrices [31,32,33,34] have postulated paracrine effects in addition to the ability of transplanted cardiomyocytes to directly supplement the beating of the heart. However, what these paracrine effects are and how they influence the cardiac function directly has been a bone of contention for a long time. While several studies have shown that immunomodulation is one of the primary effects of MSC transplantation [7], this possibility has not been given much thought with respect to cardiomyocytes owing to their allure of potential beating activity. Our results show that the immune system is an important mediator through which cardiomyocytes are able to improve cardiac function. However, how the altered immune response affects the engraftment and potential differentiation of the transplanted cells was not carried out in this study. This information is essential in order to understand the contribution of the paracrine effects and the direct benefit of the engrafted cells. These experiments will also help to improve the survival and electromechanical coupling of the transplanted cells, thereby alleviating any arrhythmogenic effects arising from cell transplantation. These questions are highly relevant for clinical translation and should be resolved to avoid the tortuous mistakes made in the development and testing of bone-marrow derived cell therapies, which were invariably tested using immune-deficient mice models and eventually failed to translate in the clinical setting where patients had a different immune status and noncomparable response.

Based on our transcriptomic analysis, the beneficial effects of cardiomyocyte transplantation appear to manifest primarily through its effect on circadian regulation and its epigenetic modification through histone modification thereby altering the mitochondrial metabolism in the heart and blood. It has been known for a long time that the heart has its own intrinsic peripheral circadian clock [35] and that the circadian clock within the cardiomyocyte influences its metabolism [36]. There is an interplay between the circadian rhythm, metabolism and physiology [37]. It is also known that the immune response is also highly influenced by the circadian rhythm [38]. In particular, Ly6C^hi^ inflammatory monocytes are highly regulated by variations in circadian gene Bmal1, otherwise known also as ARNTL [39]. This also opens up new potential therapeutic opportunities for modifying the cardiomyocyte circadian clock genes to improve cardiac function. Neonatal cardiomyocytes [40] and ESC derived cardiomyocytes [41] have been shown to exhibit a fully developed circadian rhythm with undifferentiated ESC showing no rhythmicity. However, it is important to note that further experiments are needed to confirm the cell dependent effects observed in this study. As shown by Vagnozzi et al. [42] with adult stem cells, it could be that a similar response could be induced by injecting dead cells or eliciting the innate immune response. This knowledge might also help in choosing the optimal cell type for cardiac cell therapy with the transplantation of cells eliciting a similar immune response leading to a potential improvement in cardiac function. The absence of increased capillaries and infarct area reduction observed in our work suggests that cardiomyocyte transplantation alone might not be sufficient to completely repair and regenerate the infarcted heart, and the influence of cell engraftment on these processes should be further studied.

## Figures and Tables

**Table 1:** List of antibodies used.

Target	Clone	Source
CD45	30-F11	Biolegend
CD11b	M1/70	Biolegend
CD11c	N418	Biolegend
NK1.1	PK136	Biolegend
Ly6G	1A8	Biolegend
Ly6C	Hk1.4	Biolegend
CCR2	475301	R and D
MHC II	AF6-120.1	Biolegend
CD3e	145-2C11	Biolegend
CD8a	53-6.7	Biolegend
CD4	RM4-5	Biolegend
FoxP3	MF-14	Biolegend
Anti-CD31	MEC 7.46	Abcam
Anti-CD68	FA-11	Invitrogen
Anti-GFP	Rabbit polyclonal	Abcam
